# Trends of hospitalisations rates in a cohort of HIV-infected persons followed in an Italian hospital from 1998 to 2016

**DOI:** 10.1017/S0950268819000098

**Published:** 2019-02-22

**Authors:** S. Bellino, A. Borghetti, F. Lombardi, L. Camoni, A. Ciccullo, G. Baldin, S. Belmonti, D. Moschese, S. Lamonica, R. Cauda, P. Pezzotti, S. Di Giambenedetto

**Affiliations:** 1 Department of Infectious Diseases, Istituto Superiore di Sanità, Viale Regina Elena 299, 00161 Rome, Italy; 2 Fondazione Policlinico Universitario A. Gemelli IRCCS, Roma Italia, UOC Malattie Infettive; 3 Università Cattolica del Sacro Cuore, Roma Italia, Istituto di Clinica Malattie Infettive; 4 National Centre of Health Technology Assessment, Istituto Superiore di Sanità, Viale Regina Elena 299, 00161 Rome, Italy

**Keywords:** Causes for hospitalisation, cohort study, HIV, hospitalisation rates, multiple failure-time data analysis, Poisson regression model

## Abstract

Here we evaluated hospitalisation rates and associated risk factors of human immunodeficiency virus (HIV)-infected individuals who were followed up in an Italian reference hospital from 1998 to 2016. Incidence rates (IR) of hospitalisations were calculated for five study periods from 1998 to 2016. The random-effects Poisson regression model was used to assess risk factors for hospitalisation including demographic and clinical characteristics. To consider that more events may occur for the same subject, multiple failure-time data analysis was also performed for selected causes using the Cox proportional hazards model. We evaluated 2031 patients. During 13 173 person-years (py) of follow-up, 3356 hospital admissions were carried out for 756 patients (IR: 255 per 1000 py). IR decreased significantly over the study period, from 634 in 1998–2000 to 126 per 1000 py in 2013–2016. Major declines were detected for AIDS-defining events, non-HIV/AIDS-related infections and neurological diseases. Older age, female sex, longer HIV duration and HCV coinfection were associated with a higher hospitalisation risk, whereas higher CD4 nadir and antiretroviral therapy were associated with a reduced risk. Influence of advanced HIV disease markers declined over time. Hospitalisation rates decreased during the study period in most causes. The relative weight of hospitalisations for non-AIDS-related tumours, cardiovascular, respiratory and kidney diseases increased during the study period, whereas those for AIDS-defining events declined.

## Introduction

The widespread availability of antiretroviral therapy (ART) has led to a dramatic decrease in morbidity and mortality due to human immunodeficiency virus (HIV) infection. Indeed, a declining rate of hospitalisation in people living with HIV/AIDS and a significant reduction in the length of hospital stay shortly after the introduction of ART has already been described in previous works [1–5]. The use of ART in accordance with treatment guidelines has transformed the HIV disease into a treatable and chronic infection by suppressing HIV viral load (VL) and by improving immune status, thus resulting in a decrease in the incidence of AIDS-defining events, improvement of survival rates and an increase in life expectancy [6, 7]. However, in the late ART era some have suggested that this scenario has been shifting towards stability or even a novel increase in the rate of hospital admissions, particularly for non-AIDS-related conditions [8–11]. Perhaps this transition can be explained by the occurrence of a still high rate of late presenters, an increase in age-related disorders, toxic effects due to prolonged exposure to ART, emergence of non-AIDS-related malignancies or other co-infections and lifestyle-related factors, such as illicit drug abuse, smoking and alcohol consumption. Monitoring the hospitalisations of HIV-infected people has an important role in evaluating healthcare costs and could be indicative of the quality of healthcare assistance. Moreover, the rate and mainly the emerging causes of hospitalisation are also crucial in order to forecast the co-morbidity pattern and to develop strategies for managing persons with HIV.

In the present study, trends and causes of hospitalisation over the last two decades, i.e. 1998–2016, were evaluated in HIV-infected persons receiving care at the University Hospital ‘A. Gemelli Polyclinic Foundation’ in Rome, Italy. Because of increasing evidence of the role of age-related comorbidities in the risk of hospitalisation, the impact of demographic and clinical factors on hospital admission rates was also assessed.

## Methods

A retrospective cohort study was conducted to include all HIV-infected individuals who had received specialised care between 1998 and 2016 in a large (1550 beds) tertiary referral Italian hospital, ‘A. Gemelli’ Polyclinic, located in Rome, Italy, which provides specialist care for persons who have HIV. The hospital also includes all clinical and surgical wards as well as outpatient facilities. A large number of people living with HIV are followed-up at the Infectious Disease outpatient service of ‘A. Gemelli’ Polyclinic (one of the regional referral centres for HIV), where follow-up visits are usually scheduled every 3 months. Of note, access to care is free and direct contact with the treating clinicians, who can establish elective admissions to the Infectious Disease ward, is guaranteed for every patient. Acute events requiring immediate hospitalisation are managed through patients’ admissions to the emergency room and subsequent transfer to the pertinent ward. HIV-infected patients followed-up at ‘A. Gemelli’ Polyclinic and admitted in other hospitals (especially small clinical centres) during emergencies are usually transferred to ‘A. Gemelli’ after their haemodynamic parameters have stabilised.

The study was approved by the local Institutional Ethics Committee (study protocol n. 10978/15). Data were collected using the medical records database, which was established in 1998 and which is administered by a data manager and accessible only to clinicians and technical staff. All subjects included in the study provided written informed consent. Demographic characteristics and clinical information derived from various clinical departments were merged into a single database. Morbidity data associated with hospitalisation were classified according to the ‘International Classification of Diseases, 9th Revision, Clinical Modification’ (ICD-9-CM). Core variables included age, gender, date of first positive HIV test, HIV exposure category, hepatitis B and C virus serostatus, ART history and regimens, current and nadir CD4 T-cell counts, HIV VL and categories of hospitalisation diagnoses.

The objective of the study was to evaluate the trend of hospitalisation rates over the last 19 years by study period and diagnostic categories and to assess risk factors for hospitalisation. Number of hospitalisations, person-years (py) at risk and incidence rates (IR) of hospitalisation (per 1000 py of follow-up) were calculated overall and for five study periods: 1998–2000, 2001–2004, 2005–2008, 2009–2012 and 2013–2016. The follow-up period, starting from the beginning of data recording in our centre (1998), was divided every three or four calendar years by considering major changes in HIV treatment guidelines, such as the introduction of new drugs and/or new paradigms of care. All ordinary hospital admissions (i.e. excluding day-hospital and home health care) were considered and patients contributed py at risk for periods they were not hospitalised, starting from the date of their first clinical record up to their last follow-up visit (patients without death declaration who did not undergo blood sampling for more than 1 year were considered as lost to follow-up). The average length of stay was calculated by considering the number of days spent in the hospital for each admission. The first hospitalisation was excluded when it occurred the same day as access to care; patients without follow-up were also excluded. In the most recent study period, for the non-hospitalised patients, follow-up closed on 31 December 2016, or 6 months after the last visit if it occurred before June 2016.

Reasons for hospitalisation were determined from the discharge reports. Analyses were performed for any reason for hospitalisation and for 10 non-mutually exclusive causes, classified by major diagnostic groups: AIDS-defining events, infections not directly related to HIV infection, non-AIDS-related tumours, cardiovascular, respiratory, kidney/urinary tract diseases, neurological, neuropsychiatric disorders, skin and subcutaneous tissue diseases and other conditions. Diagnostic groups were defined according to the principles listed in the ICD-9-CM, but also considering separately all clinical conditions that could have been associated with HIV infection (particularly AIDS-defining events, HIV-related tumours).

Comparisons of rates across study periods for each cause of hospital admission were assessed using the univariable Poisson regression model clustered on person (clustered sandwich estimator), which adjusts for within-patient correlation. In addition to IR, the relative weight over time of each specific reason for hospital admission was also examined. This was calculated as percentage per period of hospitalisations due to each specific diagnostic group with respect to all hospitalisations in the same period. Moreover, the multivariable Poisson regression model with random subject effect, to account for within-subject dependency, was used to assess risk factors for hospitalisation. These included both demographic and clinical characteristics, such as age class, gender, HIV exposure category, years since first positive HIV test, hepatitis B or C virus coinfection, CD4 nadir, current CD4 levels, HIV VL and use of ART; we also considered interaction terms among previous factors and time periods. Covariates with a *P*-value <0.10 in the univariable analysis entered into the multivariable model. For this analysis, risk factors were updated for each time period or hospitalisation; therefore, the last recorded value (i.e. within the previous 6 months) of covariates was considered. Finally, to take into account that more events might occur for the same subject, Cox regression analysis of multiple failure-time data was also performed [12], stratifying by four selected causes of hospitalisation, AIDS-defining diseases, non-HIV/AIDS-related infections, non-AIDS-related tumours and cardiovascular diseases. Specifically, an extension of the original Cox model was applied to take into account that more events of different types might occur for the same subject, as in the case of admissions to hospital due to different clinical diagnoses. The Cox model was fitted with the sandwich estimator, clustering on subject, and stratifying on each type of event (stratum). The model ignores the ordering of events and treats each failure occurrence as belonging in an independent stratum. All subjects are at risk for all events, and when a subject experiences one of the events he remains at risk for all others. Therefore, if there are *k* possible events, each subject will appear *k* times in the dataset, once for each possible failure.

Statistical analyses were performed using Stata software, version 13 (Stata Cooperation, College Station, Texas, USA).

## Results

### Demographic and clinical characteristics at baseline

During the study period, i.e. 1998–2016, a total of 2031 patients were followed-up, with a median length of follow-up equal to 4.8 years. According to our definition, 936/2031 (46%) patients were considered lost to follow-up.

Median age was 37 years (interquartile range (IQR) 31–44), 68.9% were males, 72.9% were Italians and 41.2% reported heterosexual contact as probable HIV exposure (Table 1). For most participants (75.1%), the time since the first positive HIV test was ⩽4 years, 65.7% resulted naïve to ART, 2.8% were co-infected with hepatitis B and 11.2% with hepatitis C virus (HCV). Among injection drug users, 44.8% of patients were co-infected with HCV; for all the other exposure categories, the percentage of positive subjects was 5.3%.

**Table 1.** Demographic and clinical characteristics at the beginning of each study period

	All periods	1998–2000	2001–2004	2005–2008	2009–2012	2013–2016
Total patients, *n*	2031	410	828	1038	1117	1159
Person-years	11 173	634	2119	2269	3606	3844
Follow-up (years), median (IQR)	4.8 (1.8–10.4)	1.5 (0.9–2.3)	2.9 (1.2–4.0)	3.7 (1.7–4.0)	4.0 (2.5–4.0)	4.0 (2.9–4.0)
Class of age (years), *n* (%)						
<35	811 (40.0)	180 (43.9)	304 (36.7)	319 (30.7)	268 (24.0)	229 (19.8)
35–44	760 (37.4)	165 (40.2)	382 (46.1)	446 (43.0)	428 (38.3)	358 (30.9)
45–54	312 (15.4)	47 (11.5)	97 (11.7)	194 (18.7)	288 (25.8)	373 (32.2)
** ⩾**55	147 (7.2)	18 (4.4)	45 (5.4)	79 (7.6)	133 (11.9)	198 (17.1)
Age (years), median IQR	37.4 (31.5–44.2)	36.2 (31.9–40.9)	37.5 (32.3–42.5)	39.9 (33.8–45.4)	42.5 (35.3–48.4)	44.8 (37.2–51.7)
Gender, *n* (%)						
Male	1399 (68.9)	257 (62.7)	535 (64.6)	678 (65.3)	752 (67.3)	824 (71.1)
Female	633 (31.1)	153 (37.3)	293 (35.4)	360 (34.7)	365 (32.7)	335 (28.9)
Nationality, *n* (%)						
Italian	1480 (72.9)	337 (82.2)	617 (74.5)	779 (75.0)	859 (76.9)	893 (77.0)
Other	530 (26.1)	67 (16.3)	203 (24.5)	251 (24.2)	254 (22.7)	257 (22.2)
Not reported	21 (1.0)	6 (1.5)	8 (1.0)	8 (0.8)	4 (0.4)	9 (0.8)
HIV exposure, *n* (%)						
Heterosexual contact	836 (41.2)	179 (43.7)	389 (47.0)	500 (48.0)	528 (47.0)	496 (43.0)
Homosexual contact	722 (35.5)	83 (20.2)	216 (26.1)	334 (32.2)	412 (36.9)	482 (41.6)
Injection drug use	306 (15.1)	138 (33.7)	178 (21.5)	147 (14.2)	115 (10.3)	95 (8.2)
Not reported	167 (8.2)	10 (2.4)	45 (5.4)	57 (5.5)	62 (5.5)	86 (7.4)
Hepatitis B virus coinfection, *n* (%)						
Positive	57 (2.8)	13 (3.2)	35 (4.2)	38 (3.7)	34 (3.0)	28 (2.0)
Negative	1974 (97.2)	397 (96.8)	793 (95.8)	1000 (96.3)	1083 (97.0)	1131 (98.0)
Hepatitis C virus coinfection, *n* (%)						
Positive	228 (11.2)	57 (13.9)	151 (18.2)	163 (15.7)	146 (13.1)	114 (9.8)
Negative	1803 (88.8)	353 (86.1)	677 (81.8)	875 (84.3)	971 (86.9)	1045 (90.2)
Years since first positive HIV test, *n* (%)						
0–4	1525 (75.1)	273 (66.6)	568 (68.6)	592 (57.0)	495 (44.3)	411 (35.5)
5–10	210 (10.3)	71 (17.3)	126 (15.2)	268 (25.8)	360 (32.2)	340 (29.3)
>10	296 (14.6)	66 (16.1)	134 (16.2)	178 (17.2)	262 (23.5)	408 (35.2)
CD4 nadir (classes), *n* (%)						
<200	835 (41.1)	184 (44.9)	398 (48.1)	511 (49.2)	553 (49.5)	559 (48.3)
200–349	474 (23.3)	106 (25.8)	217 (26.2)	271 (26.1)	319 (28.6)	330 (28.5)
** ⩾**350	718 (35.3)	119 (29.0)	212 (25.6)	256 (24.7)	245 (21.9)	268 (23.1)
Missing	4 (0.2)	1 (0.2)	1 (0.1)	0 (0.0)	0 (0.0)	2 (0.2)
CD4 nadir (cells/μl), median IQR	259 (103–428)	238 (99–379)	215 (74–354)	202 (76–343)	202 (76–326)	207 (76–330)
CD4 + T-cell counts/μl (classes), *n* (%)						
<200	676 (33.3)	165 (40.2)	256 (30.9)	206 (19.8)	132 (11.8)	110 (9.5)
200–349	451 (22.2)	103 (25.1)	200 (24.1)	247 (23.8)	178 (15.9)	140 (12.1)
350–499	393 (19.3)	70 (17.1)	173 (20.9)	252 (24.3)	273 (24.4)	241 (20.8)
** ⩾**500	507 (25.0)	71 (17.3)	198 (23.9)	333 (32.1)	534 (47.8)	666 (57.5)
Missing	4 (0.2)	1 (0.2)	1 (0.1)	0 (0.0)	0 (0.0)	2 (0.2)
CD4 + T-cell counts (cells/μl), median IQR	313 (142–500)	259 (112–442)	323 (151–490)	385 (234–554)	484 (325–646)	546 (378–748)
HIV viral load copies/ml (classes), *n* (%)						
⩽50	367 (18.1)	40 (9.8)	213 (25.7)	448 (43.2)	690 (61.8)	842 (72.6)
51–10 000	525 (25.8)	125 (30.5)	210 (25.4)	239 (23.0)	182 (16.3)	131 (11.3)
>10 000	1128 (55.5)	241 (58.8)	401 (48.4)	349 (33.6)	245 (21.9)	185 (16.0)
Missing	11 (0.5)	4 (1.0)	4 (0.5)	2 (0.2)	0 (0.0)	1 (0.1)
HIV viral load (log_10_ copies/ml), median IQR	4.2 (2.7–5.0)	4.3 (3.2–5.0)	3.9 (1.7–4.9)	2.7 (1.7–4.4)	1.7 (1.7–3.7)	0.8 (0.0–2.0)
Antiretroviral therapy, *n* (%)						
Naive	1334 (65.7)	273 (66.6)	404 (48.8)	413 (39.8)	308 (27.6)	212 (18.3)
Only NRTIs	67 (3.3)	37 (9.0)	30 (3.6)	94 (9.1)	74 (6.6)	31 (2.7)
2 NRTI + NNRTI	192 (9.4)	18 (4.4)	158 (19.1)	228 (22.0)	262 (23.5)	314 (27.1)
2 NRTIs + PI (boosted/unboosted)	374 (18.4)	75 (18.3)	215 (26.0)	270 (26.0)	428 (38.3)	386 (33.3)
Combinations including integrase inhibitor	12 (0.6)	0 (0.0)	0 (0.0)	1 (0.1)	12 (1.1)	26 (2.2)
Other combinations	52 (2.6)	7 (1.7)	21 (2.5)	32 (3.1)	33 (2.9)	190 (16.4)

All characteristics refer to the beginning of each study period (for CD4 + T cell and HIV viral load, the most recent values within the previous 6 months were considered).

At enrolment, a large proportion of individuals had CD4 T-cell counts <200 (33.3%) and HIV VL >10 000 copies (55.5%). The median values were 313 cells/μl (IQR 142–500) and 4.2 log_10_ copies/ml (IQR 2.7–5.0) for CD4 counts and HIV VL, respectively. Characteristics of the patients over the study period showed an increase in the median age and of the time since the HIV diagnosis, an improvement in HIV disease markers (CD4 and HIV VL) and a change in the ART regimen type (Table 1). The number of treatment-naive subjects declined over time because of clinical disease progression during the follow-up period and also because, according to several updates of the Italian guidelines for ART initiation, the recommended CD4 T-cell count for starting therapy increased from <350 to <500 cells/μl.

### Rates and reasons for hospitalisations

In 13 173 py of follow-up, 3356 hospital admissions took place, resulting in an IR of 255 per 1000 py (95% confidence interval (CI) 246–264); 756 patients were hospitalised at least once (Table 2). The median inpatient length of stay was 6 days (IQR 4–11) and remained stable over the entire period. Details on 1998–2016 hospitalisations in each diagnostic category are shown in Supplementary Table S1. The highest number of hospitalisations was for AIDS-defining conditions, non-HIV/AIDS-related infections and cardiovascular diseases; in particular, AIDS-related tumours represented 51% of the AIDS-defining events. The overall rate of hospitalisation decreased significantly over the years (*P* < 0.0001), from 634 (95% CI 575–699) in 1998–2000 to 126 (95% CI 115–138) in 2013–2016. AIDS-defining events, cardiovascular diseases and non-AIDS-related tumours had the highest hospitalisation rates, 34 and 31 per 1000 py respectively, followed by psychiatric disorders (28 per 1000 py) and respiratory tract disorders (20 per 1000 py). Hospital admission rates significantly decreased over time for all major diagnostic groups except for cardiovascular and kidney diseases (Table 2). Moreover, for non-AIDS-related tumours as well as skin and subcutaneous tissue illnesses, a significant decrease was observed only in the last calendar period. Major declines were detected in the 2013–2016 period as compared with the 1998–2000 period for psychiatric disorders (IR ratio (IRR) 0.04), AIDS-defining events (IRR 0.10), neurological diseases (IRR 0.13) and non-HIV/AIDS-related infections (IRR 0.17). In the last period most of the hospitalisations were for cardiovascular diseases (IR 29 per 1000 py), AIDS-defining events (IR 23 per 1000 py) and non-HIV/AIDS-related infections (IR 22 per 1000 py) (Table 2). The relative weight of hospitalisations for non-HIV/AIDS-related infections, neurological and skin diseases did not change from 1998 to 2016: it declined for AIDS-defining events and psychiatric disorders and rose for non-AIDS-related tumours, cardiovascular, respiratory and kidney diseases (Table 2).

**Table 2.** Hospitalisation rates, overall and for major diagnostic groups per period

Causes of hospitalisation	Hospitalised patients	Number of hospitalisations	Relative weights (%)	IR × 1000 (95% CI)	IRR (95% CI)	*P*-value	*χ*^2^ test^a^
Total hospitalisations	756	3356		255 (246–264)			
All causes							
1998–2000	166	402	–	634 (575–699)	1 (ref)		
2001–2004	334	964	–	455 (427–485)	0.72 (0.57–0.90)	***0.0049***	
2005–2008	345	857	–	289 (270–309)	0.45 (0.36–0.58)	***<0.0001***	
2009–2012	275	648	–	180 (166–194)	0.28 (0.22–0.36)	***<0.0001***	
2013–2016	234	485	–	126 (115–138)	0.20 (0.15–0.26)	***<0.0001***	***<0.0001***
AIDS-defining diseases							
1998–2000	46	150	31.3	236 (201–277)	1 (ref)		
2001–2004	110	387	33.5	183 (165–202)	0.77 (0.49–1.22)	*0.2689*	
2005–2008	94	243	22.1	82 (72–93)	0.35 (0.22–0.55)	***<0.0001***	
2009–2012	48	138	16.6	38 (32–45)	0.16 (0.10–0.28)	***<0.0001***	
2013–2016	43	90	16.2	23 (19–29)	0.10 (0.05–0.18)	***<0.0001***	***<0.0001***
Non-HIV/AIDS-related infections							
1998–2000	53	82	17.1	129 (104–161)	1 (ref)		
2001–2004	150	289	25	136 (122–153)	1.05 (0.70–1.59)	*0.7964*	
2005–2008	117	230	20.9	78 (68–88)	0.60 (0.39–0.92)	***0.0189***	
2009–2012	100	169	20.3	47 (40–55)	0.36 (0.23–0.57)	***<0.0001***	
2013–2016	59	85	15.3	22 (18–27)	0.17 (0.11–0.28)	***<0.0001***	***<0.0001***
Non-AIDS-related tumours							
1998–2000	15	32	6.7	50 (36–71)	1 (ref)		
2001–2004	31	97	8.4	46 (38–56)	0.91 (0.40–2.07)	*0.8175*	
2005–2008	38	109	9.9	37 (30–44)	0.73 (0.32–1.65)	*0.4477*	
2009–2012	46	109	13.1	30 (25–37)	0.60 (0.27–1.33)	*0.2089*	
2013–2016	41	67	12.1	17 (14–22)	0.34 (0.16–0.75)	***0.0074***	***0.0035***
Cardiovascular and other circulatory diseases							
1998–2000	14	25	5.2	39 (27–58)	1 (ref)		
2001–2004	30	64	5.5	30 (24–39)	0.77 (0.34–1.73)	*0.5218*	
2005–2008	58	135	12.3	46 (38–54)	1.15 (0.54–2.46)	*0.7104*	
2009–2012	61	111	13.3	31 (26–37)	0.78 (0.38–1.61)	*0.5046*	
2013–2016	64	110	19.8	29 (24–35)	0.73 (0.34–0.53)	*0.4024*	*0.3036*
Psychiatric disorders							
1998–2000	31	76	15.9	120 (96–150)	1 (ref)		
2001–2004	48	114	9.9	54 (45–65)	0.45 (0.26–0.77)	***0.0035***	
2005–2008	41	105	9.5	35 (29–43)	0.29 (0.15–0.57)	***0.0003***	
2009–2012	37	55	6.6	15 (12–20)	0.13 (0.07–0.23)	***<0.0001***	
2013–2016	17	21	3.8	6 (4–8)	0.04 (0.02–0.09)	***<0.0001***	***<0.0001***
Neurological diseases							
1998–2000	21	39	8.1	62 (45–84)	1 (ref)		
2001–2004	37	66	5.7	31 (25–40)	0.51 (0.30–0.84)	***0.0092***	
2005–2008	36	74	6.7	25 (20–31)	0.40 (0.19–0.85)	***0.0174***	
2009–2012	21	39	4.7	11 (8–15)	0.18 (0.08–0.38)	***<0.0001***	
2013–2016	23	32	5.8	8 (6–12)	0.13 (0.07–0.27)	***<0.0001***	***<0.0001***
Respiratory tract diseases							
1998–2000	20	24	5	38 (25–56)	1 (ref)		
2001–2004	32	39	3.4	18 (13–25)	0.49 (0.27–0.89)	***0.0188***	
2005–2008	47	78	7.1	26 (21–33)	0.69 (0.36–1.35)	*0.2808*	
2009–2012	38	51	6.1	14 (11–19)	0.37 (0.21–0.67)	***0.0011***	
2013–2016	43	72	13	19 (15–24)	0.49 (0.22–1.09)	*0.0809*	***0.0116***
Kidney and urinary tract diseases							
1998–2000	9	14	2.9	22 (13–37)	1 (ref)		
2001–2004	24	28	2.4	13 (9–19)	0.60 (0.26–1.39)	*0.2349*	
2005–2008	30	41	3.7	14 (10–19)	0.63 (0.27–1.45)	*0.276*	
2009–2012	42	78	9.4	22 (17–27)	0.98 (0.43–2.23)	*0.9623*	
2013–2016	26	37	6.7	10 (7–13)	0.44 (0.19–1.02)	*0.0546*	***0.0359***
Skin and subcutaneous tissue diseases							
1998–2000	4	8	1.7	13 (6–25)	1 (ref)		
2001–2004	11	13	1.1	6 (4–11)	0.49 (0.12–2.05)	*0.3255*	
2005–2008	13	17	1.5	6 (4–9)	0.45 (0.11–1.89)	*0.2783*	
2009–2012	11	12	1.4	3 (2–6)	0.26 (0.06–1.10)	*0.0681*	
2013–2016	9	11	2	3 (2–5)	0.23 (0.06–0.92)	*0.0380*	*0.1239*
Other							
1998–2000	18	29	6.1	46 (32–66)	1 (ref)		
2001–2004	43	59	5.1	28 (22–36)	0.61 (0.33–1.11)	*0.1067*	
2005–2008	48	69	6.3	23 (18–29)	0.51 (0.28–0.92)	***0.0267***	
2009–2012	43	71	8.5	20 (16–25)	0.43 (0.23–0.81)	***0.0089***	
2013–2016	21	30	5.4	8 (5–11)	0.17 (0.08–0.36)	***<0.0001***	***0.0001***

CI, confidence interval; IR, incidence rate, IRR, incidence rate ratio; IRR and *P*-value were estimated using the Poisson regression model.

Reasons for hospitalisation were non-mutually exclusive. Relative weights were calculated as percentage per period of each cause of hospitalisation *vs*. overall.

‘Other’ category includes signs and symptoms not classified elsewhere and factors influencing health status; three hospitalisations due to ear-nose-mouth, haematological, endocrine-metabolic, gastrointestinal and musculoskeletal diseases were also included. Bold italic values represent statistically significant *p*-values.

a Wald *χ*^2^ test statistics is the significance test for the model.

For a more comprehensive analysis, Supplementary Table S2 shows the IRR using the random-effects Poisson regression model, which seems more powerful for detecting differences across study periods as compared with the Poisson model with clustered on patient.

### Risk factors for hospitalisation

In the multivariable model, older age (⩾55 years compared with <35, relative risk (RR) 2.39), female sex (RR 1.48), longer time from infection (5–10 (RR 1.27), ⩾10 (RR 1.77) compared with 0–4 years) and HCV coinfection (RR 1.29) were associated with a higher risk of hospitalisation; by contrast, higher CD4 nadir (200–349 (RR 0.80), ⩾350 (RR 0.53) compared with <200) and ART use (RR 0.67 *vs.* naive) were associated with a reduced risk of hospitalisation (Table 3). Moreover, an interaction effect between CD4 counts, HIV VL and time periods was observed, indicating that the influence of advanced HIV disease markers on the risk of hospital admission declined over time. Specifically, at the same levels of CD4 (<200, 200–349, 350–499, ⩾500 cells/μl) and VL (⩽50, >50 copies/ml) risk of hospitalisation declined quite continuously during the study period. Given the relationship between ART use and CD4 and VL levels, Supplementary Tables S3a and S3b report two additional models, one including only ART effect (without CD4 and VL) and one including only CD4 and VL effects (without ART). Comparing the full model with the latter two models, a higher decrease in the hospitalisation risk associated with ART use was detected (0.47 *vs.* 0.67), whereas very similar effects of CD4 and VL levels were found.

**Table 3.** Multivariable analysis of factors associated with hospitalisation – Poisson regression model

	All hospitalisations	
Factors	RR (95% CI)	*P*-value
Class of age (years)		
<35	1 (ref)	
35–44	1.11 (0.96–1.29)	*0*.*1606*
45–54	1.20 (0.99–1.46)	*0.0646*
⩾55	2.39 (1.83–3.11)	***<0.0001***
Gender		
Male	1 (ref)	
Female	1.48 (1.17–1.88)	***0.0012***
HIV exposure		
Heterosexual contact	1 (ref)	
Homosexual contact	1.03 (0.80–1.32)	*0.8330*
Injection drug use	1.30 (0.98–1.73)	*0.0708*
Not reported	2.65 (1.80–3.92)	***<0.0001***
Years since first positive HIV test		
0–4	1 (ref)	
5–10	1.27 (1.13–1.44)	***0.0001***
>10	1.77 (1.49–2.10)	***<0.0001***
Hepatitis C virus coinfection	1.29 (1.08–1.54)	***0.0049***
CD4 nadir (cells/μl)		
<200	1 (ref)	
200–349	0.80 (0.66–0.97)	***0.0259***
⩾350	0.53 (0.42–0.67)	***<0.0001***
Antiretroviral therapy		
Naïve	1 (ref)	
On ART	0.67 (0.58–0.76)	***<0.0001***
CD4 (cells/μl), HIV VL (copies/ml), period		
** CD4 <200, HIV VL >50, 1998–2000**	1 (ref)	
CD4 <200, VL >50, 2001–2004	1.34 (1.04–1.73)	***0.0244***
CD4 <200, VL >50, 2005–2008	0.98 (0.73–1.32)	*0.8806*
CD4 <200, VL >50, 2009–2012	0.57 (0.39–0.82)	***0.0028***
CD4 <200, VL >50, 2013–2016	0.49 (0.31–0.78)	***0.0025***
** CD 4 200–349, HIV VL >50, 1998–2000**	1 (ref)	
CD 4 200–349, VL >50, 2001–2004	0.57 (0.40–0.83)	***0.0031***
CD 4 200–349, VL >50, 2005–2008	0.55 (0.38–0.81)	***0.0024***
CD4 200–349, VL >50, 2009–2012	0.25 (0.16–0.40)	***<0.0001***
CD4 200–349, VL >50, 2013–2016	0.15 (0.08–0.27)	***<0.0001***
** CD4 350–499, HIV VL >50, 1998–2000**	1 (ref)	
CD4 350–499, VL >50, 2001–2004	1.07 (0.67–1.71)	*0.7742*
CD4 350–499, VL >50, 2005–2008	0.91 (0.56–1.47)	*0.6886*
CD4 350–499, VL >50, 2009–2012	0.39 (0.22–0.68)	***0.001***
CD4 350–499, VL >50, 2013–2016	0.25 (0.13–0.49)	***<0.0001***
** CD4 ⩾500, HIV VL >50, 1998–2000**	1 (ref)	
CD4 **⩾**500, VL >50, 2001–2004	0.55 (0.36–0.83)	***0.0050***
CD4 **⩾**500, VL >50, 2005–2008	0.28 (0.18–0.44)	***<0.0001***
CD4 **⩾**500, VL >50, 2009–2012	0.23 (0.14–0.38)	***<0.0001***
CD4 **⩾**500, VL >50, 2013–2016	0.10 (0.06–0.17)	***<0.0001***
** CD4 <200, HIV VL** ⩽**50, 1998–2000**	1 (ref)	
CD4 <200, VL ⩽50, 2001–2004	0.62 (0.41–0.93)	***0.0215***
CD4 <200, VL ⩽50, 2005–2008	0.30 (0.19–0.47)	***<0.0001***
CD4 <200, VL ⩽50, 2009–2012	0.20 (0.12–0.33)	***<0.0001***
CD4 <200, VL ⩽50, 2013–2016	0.31 (0.18–0.52)	***<0.0001***
** CD4 200–349, HIV VL** ⩽**50, 1998–2000**	1 (ref)	
CD4 200–349, VL ⩽50, 2001–2004	0.31 (0.19–0.51)	***<0.0001***
CD4 200–349, VL ⩽50, 2005–2008	0.18 (0.11–0.29)	***<0.0001***
CD4 200–349, VL ⩽50, 2009–2012	0.16 (0.10–0.26)	***<0.0001***
CD4 200–349, VL ⩽50, 2013–2016	0.08 (0.05–0.14)	***<0.0001***
** CD4 350–499, HIV VL** ⩽**50, 1998–2000**	1 (ref)	
CD4 350–499, VL ⩽50, 2001–2004	0.79 (0.35–0.80)	*0.5749*
CD4 350–499, VL ⩽50, 2005–2008	0.63 (0.28–1.40)	*0.2585*
CD4 350–499, VL ⩽50, 2009–2012	0.53 (0.24–1.18)	*0.1199*
CD4 350–499, VL ⩽50, 2013–2016	0.36 (0.16–0.82)	***0.0145***
** CD4** ⩾**500, HIV VL** ⩽**50, 1998–2000**	1 (ref)	
CD4 ⩾500, VL ⩽50, 2001–2004	0.29 (0.15–0.56)	***0.0002***
CD4 ⩾500, VL ⩽50, 2005–2008	0.23 (0.12–0.42)	***<0.0001***
CD4 ⩾500, VL ⩽50, 2009–2012	0.13 (0.07–0.25)	***<0.0001***
CD4 ⩾500, VL ⩽50, 2013–2016	0.10 (0.05–0.19)	***<0.0001***

Bold italic values represent statistically significant *p*-values.

Multiple failure-time data analysis was performed to take into account that more events (AIDS-defining diseases, non-HIV/AIDS-related infections, non-AIDS-related tumours and cardiovascular diseases) might occur in the same subject. This analysis showed different effects of the examined factors on four selected causes of hospitalisation (Table 4). Hospital admission due to non-AIDS-related tumours and cardiovascular diseases was predicted by older age, particularly for ⩾55 year olds (hazard ratio (HR) 5.31 and 5.28, respectively) and by HIV exposure category. The male homosexual contacts category was associated with non-AIDS-related tumours (HR 1.73) and injecting drug use to cardiovascular diseases (HR 2.34). HCV coinfection was detrimental for hospitalisations due to non-HIV/AIDS-related infections (HR 2.89). Higher CD4 levels (⩾200 cells/μl) were beneficial for all causes, whereas a higher CD4 nadir (⩾350 cells/μl) was beneficial only for AIDS-defining events (HR 0.39). Use of ART appeared to be associated with a higher risk of hospitalisation due to AIDS-defining illnesses (HR 2.10) or non-AIDS-related tumours (HR 2.04), whereas a longer time from the first positive HIV test (HR 1.93, 5–10 *vs.* 0–4 years) was only associated with a higher risk of hospitalisation due to cardiovascular diseases. Higher VL (HIV VL >50 *vs.* ⩽50 copies/ml) was related to admissions for AIDS-defining events (HR 1.53) and non-HIV/AIDS-related infections (1.34). Finally, major declines in hospitalisations were observed in the last calendar period (2013–2016) as compared with the first one (1998–2000); these were statistically significant for AIDS-defining diseases (HR 0.43, *P* = 0.0113) and almost significant for non-AIDS-related tumours (HR 0.44, *P* = 0.0597).

**Table 4.** Analysis of multiple failure-time data – Cox proportional hazards model

Factors	AIDS-defining diseases		Non-HIV/AIDS-related infections		Non-AIDS-related tumours		Cardiovascular and other circulatory diseases	
	997		853		413		445	
Number of hospitalisations	HR (95% CI)	*P*-value	HR (95% CI)	*P*-value	HR (95% CI)	*P*-value	HR (95% CI)	*P*-value
Class of age (years)								
<35	1 (ref)		1 (ref)		1 (ref)		1 (ref)	
35–44	1.28 (0.89–1.85)	*0*.*1887*	0.98 (0.73–1.31)	*0*.*8763*	2.52 (1.21–5.21)	***0***.***0131***	1.04 (0.46–2.34)	*0*.*9194*
45–54	1.43 (0.94–2.20)	*0.0980*	1.28 (0.94–1.75)	*0.1174*	4.75 (2.27–9.92)	***<0.0001***	1.85 (0.82–4.20)	*0.1405*
⩾55	1.33 (0.82–2.14)	*0.2420*	0.99 (0.65–1.51)	*0.9729*	5.31 (2.49–11.33)	***<0.0001***	5.28 (2.43–11.47)	***<0.0001***
Gender								
Male	1 (ref)		1 (ref)		1 (ref)		1 (ref)	
Female	0.78 (0.52–1.16)	*0.2264*	0.96 (0.72–1.29)	*0.8040*	1.06 (0.64–1.77)	*0.8108*	0.66 (0.42–1.04)	*0.0738*
HIV exposure								
Heterosexual contact	1 (ref)		1 (ref)		1 (ref)		1 (ref)	
Homosexual contact	1.30 (0.88–1.93)	*0.1880*	1.34 (0.93–1.93)	*0.1127*	1.73 (1.01–2.96)	***0.0461***	1.05 (0.62–1.80)	*0.8436*
Injection drug use	1.09 (0.66–1.79)	*0.7258*	2.16 (1.50–3.09)	***<0.0001***	0.99 (0.49–2.02)	*0.9855*	2.34 (1.16–4.73)	***0.0173***
Not reported	1.65 (1.82–3.33)	*0.1597*	1.69 (0.97–2.93)	*0.0640*	1.74 (0.65–4.60)	*0.2663*	1.73 (0.86–3.49)	*0.1268*
Years since first HIV+ test								
0–4	1 (ref)		1 (ref)		1 (ref)		1 (ref)	
5–10	1.02 (0.69–1.52)	*0.9172*	1.02 (0.75–1.37)	*0.9026*	1.74 (0.44–1.23)	*0.2438*	1.93 (1.30–2.86)	***0.0010***
>10	0.83 (0.51–1.35)	*0.4519*	0.92 (0.66–1.29)	*0.6269*	1.54 (0.95–2.52)	*0.0819*	1.71 (1.00–2.93)	*0.0512*
**Hepatitis C virus coinfection**	0.75 (0.47–1.20)	*0.2342*	2.89 (2.08–4.00)	***<0.0001***	0.86 (0.41–1.79)	*0.6894*	1.00 (0.61–1.64)	*0.9997*
CD4 nadir (cells/μl)								
<200	1 (ref)		1 (ref)		1 (ref)		1 (ref)	
200–349	1.13 (0.65–1.95)	*0.6731*	0.98 (0.70–1.36)	*0.9057*	0.79 (0.44–1.44)	*0.4425*	0.84 (0.51–1.39)	*0.5013*
⩾350	0.39 (0.21–0.74)	***0.0042***	0.92 (0.67–1.28)	*0.6317*	0.51 (0.23–1.09)	*0.0839*	0.61 (0.33–1.12)	*0.1087*
Antiretroviral therapy								
Naïve	1 (ref)		1 (ref)		1 (ref)		1 (ref)	
On ART	2.10 (1.53–2.87)	***<0.0001***	1.12 (0.86–1.46)	*0.3993*	2.04 (1.24–3.35)	***0.0049***	1.06 (0.68–1.66)	*0.7941*
CD4 + T-cell counts/μl								
<200	1 (ref)		1 (ref)		1 (ref)		1 (ref)	
200–349	0.36 (0.26–0.49)	***<0.0001***	0.62 (0.49–0.78)	***0.0001***	0.56 (0.33–0.94)	***0.0283***	0.70 (0.47–1.05)	*0.0855*
350–499	0.18 (0.11–0.28)	***<0.0001***	0.47 (0.35–0.65)	***<0.0001***	0.45 (0.24–0.81)	***0.0082***	0.56 (0.34–0.93)	***0.0267***
⩾500	0.13 (0.07–0.23)	***<0.0001***	0.39 (0.29–0.53)	***<0.0001***	0.40 (0.19–0.81)	***0.0108***	0.50 (0.27–0.90)	***0.0222***
HIV viral load copies/ml								
⩽50	1 (ref)		1 (ref)		1 (ref)		1 (ref)	
>50	1.53 (1.10–2.12)	***0.0121***	1.34 (1.02–1.75)	***0.0335***	0.92 (0.59–1.44)	*0.7284*	1.13 (0.79–1.61)	*0.5113*
Period								
1998–2000	1 (ref)		1 (ref)		1 (ref)		1 (ref)	
2001–2004	1.17 (0.76–1.78)	*0.4742*	1.53 (1.01–2.32)	***0.0461***	0.97 (0.44–2.17)	*0.9486*	1.08 (0.47–2.48)	*0.8472*
2005–2008	0.81 (0.52–1.28)	*0.3727*	1.27 (0.83–1.95)	*0.2654*	0.96 (0.42–2.20)	*0.9203*	1.94 (0.93–4.02)	*0.0758*
2009–2012	0.60 (0.33–1.09)	*0.0920*	1.15 (0.71–1.85)	*0.5614*	0.84 (0.36–2.00)	*0.7016*	1.42 (0.66–3.06)	*0.3700*
2013–2016	0.43 (0.23–0.83)	***0.0113***	0.69 (0.41–1.17)	*0.1738*	0.44 (0.18–1.03)	*0.0597*	1.27 (0.55–2.96)	*0.5780*

Bold italic values represent statistically significant *p*-values.

## Discussion

The present study showed a dramatic temporal decrease in the overall hospitalisation rate of HIV-positive patients in care from 1998 to 2016 in an Italian reference hospital located in Rome. When considering different reasons for hospitalisation, major declines were detected for AIDS-defining events, non-HIV/AIDS-related infections and neurological illnesses; by contrast, no relevant changes were observed for cardiovascular and circulatory system diseases. Moreover, minor changes were found for kidney and respiratory diseases. Therefore, cardiovascular illnesses, AIDS-defining events and non-HIV/AIDS-related infections are currently the most common reasons for hospitalisation. Of note, the relative weight of hospitalisations due to non-AIDS-related tumours, cardiovascular, respiratory and kidney diseases increased during the study period. Overall, risk of hospital admission continues to be associated with markers of advanced HIV disease (i.e. CD4 count and VL) but their influence on the risk of hospitalisation decreased dramatically, whereas ageing and HIV-associated factors had different effects on hospitalisation rates due to AIDS-defining diseases and specific non-AIDS-related conditions. Indeed, older age was associated with hospitalisations for cardiovascular and other circulatory diseases and non-AIDS-related tumours. Specifically, for cardiovascular diseases the increase was very strong not only when patients ⩾55 years were compared with individuals aged <35 years, but also when they were compared with those aged 45–54 (2.85, 95% CI 1.80–4.52). Regarding non-AIDS-related tumours, it is of note that there was a strong increase of non-AIDS-related tumours in individuals aged 45–54 years compared with those aged 35–44 years (1.89, 95% CI 1.14–3.11), whereas a slight increase was detected when patients ⩾55 years were compared with those aged 45–54 years (1.12, 95% CI 0.69–1.82). Thus, these results seem in line with those reported for the general population, where it has been found that the IR of cardiovascular diseases and cancers increase ‘exponentially’ between ages 40 and 80 [13]. This pattern is not as clear in the setting of HIV infection, where a persistent chronic inflammation that typically characterises immunological ageing is an essential contributor to several comorbidities [14]. This inflammation is particularly evident in older adults who have chronic, well-treated HIV infection, because even successful anti-viral treatment is unable to inhibit the onset of some of the complications caused by persistent immune activation. Therefore, patients with a relatively advanced age, i.e. aged >50 years, can experience pathologies that affect many older people. To confirm this, in our study, the highest risk for cardiovascular diseases and cancers was found in patients ⩾55 years of age. However, we should highlight that in this population study, patients aged ⩾55 years had a median age of only 60 years and thus did not truly represent the elderly.

In our study, higher CD4 nadir and CD4 counts, as well as ART use were associated with a reduced risk of hospitalisation. However, it is of note that when the multiple failure-time data were considered, ART use was associated with an increased risk of hospitalisation for AIDS-related events and non-AIDS-related tumours. The increased risk of tumour onset can be explained by the patients’ prolonged survival associated with ART use, whereas the relationship between ART and increased risk of AIDS-defining events remains unclear. It is possible that ART initiation could have increased the incidence of an inflammatory immune reconstitution syndrome, thus unmasking underlying infections or malignancies; however, the lack of data regarding cumulative exposure to ART at the time of hospitalisation prevents us from drawing any definite conclusions.

Of note, both CD4 count and HIV VL showed an interaction effect with time period. Indeed, more recent years played a role in reducing the risk of hospitalisation, likely because of the beneficial effect of newer antiretroviral regimens and of more comprehensive preventive strategies concerning HIV-related and non-related comorbidities. The hospitalisation rate for the ‘not reported’ HIV exposure category was higher than that for ‘heterosexual contact’ related to the other groups. However, this is a heterogeneous category for which it is difficult to find explanations.

Our findings are consistent with those of previous studies in indicating a reduction in hospital admission rates among HIV-infected patients; indeed, non-AIDS-related hospitalisations are currently more frequent than those related to AIDS, although some differences among countries were found [1, 8–11, 15–21]. The hospitalisation rate among HIV-infected persons enrolled in a large cohort in the USA slightly decreased over the study period, i.e. from 127 per 1000 py in 1999 to 102 per 1000 py in 2007 [8]. Increasing trends of hospitalisation rates for cancer and cardiovascular disease and decreasing trends for neurological disorders were observed. AIDS-defining conditions occurred at a rate of 10 admissions per 1000 py and did not significantly change over time, whereas infections accounted for the highest rate of hospitalisation [8].

In a retrospective cohort study from1999 to 2007 of people with HIV in Australia, the hospitalisation rate was 590 per 1000 py [9], which is quite in line with our data for the same period. Associated factors included prior AIDS, older age, higher HIV VL, longer duration of HIV infection and calendar year. Hospital admission rates decreased for non-opportunistic infections and non-AIDS-related tumours (from 51 to 40 per 1000 py). No relevant changes were observed for hospitalisations due to cardiovascular diseases (from 32 to 35 per 1000 py). Hospitalisation rates for persons with medically controlled HIV infection in care at 11 sites in the USA from 2005 to 2011 was 105 per 1000 py; non-AIDS-defining infections and cardiovascular diseases were the most common reason for admissions [21]. As in our study, factors associated with hospitalisation included older age, female sex, HIV exposure category, lower CD4 cell count and HCV coinfection.

Before drawing conclusions, some limits of the study should be mentioned. In particular, our findings are representative of a single Italian centre and, thus, results do not reflect the nationwide clinical scenario. An important limit could be represented by the lost to follow-up rate, considering that 46% of patients had their last visit before 2015. Moreover, some clinical variables (such as body mass index, smoking habit and alcohol use) that could be associated with hospitalisation rates due to non-AIDS-related tumours or cardiovascular diseases were not collected.

However, our work also shows important strengths. The hospital where the study was performed is a centre of reference for many clinical and surgical specialties; this allows for the observation of all medical conditions that could affect patients requiring hospitalisation, particularly HIV-infected persons. In addition, the large sample size and the long-term follow-up allowed for a broad view of changes in hospitalisation rates across different calendar periods, characterised by significant modifications in HIV treatment and prevention strategies. Moreover, mathematical models based on the ICONA Foundation Study (a large Italian cohort that includes 42 infectious disease centres) has forecast that in the next few decades, age-related comorbidities will become a substantial issue in HIV-infected individuals [22], especially due to the increased burden of cardiovascular diseases, diabetes and chronic kidney illness, which supports our findings. Finally, as this is a situation in which multiple events of different types are of interest, appropriate statistical analysis based on multiple failure events was performed, strengthening the study results.

In conclusion, our findings highlight the importance of considering non-AIDS-related conditions in the management of HIV-infected persons, focusing especially on modifiable risk factors that can affect cardiovascular diseases and implementing screening measures for non-AIDS-related tumours as well as continuing to monitor the traditional markers of advanced HIV disease. As hospital admission is an important indicator of healthcare quality, early diagnosis and entry into care are essential to further reduce hospitalisations of HIV-infected persons.

## Supplementary material

For supplementary material accompanying this paper visit https://doi.org/10.1017/S0950268819000098. click here to view supplementary material
