# Supplementary material for: Trends of hospitalisations rates in a cohort of HIV-infected persons followed in an Italian hospital from 1998 to 2016
Source: Epidemiol Infect. 2019 Feb 22;147:e89. doi: 10.1017/S0950268819000098 (PMC6518563; doi:10.1017/S0950268819000098)
Supplement: Supplementary file 1 [file S0950268819000098sup.zip › S0950268819000098sup001.doc]

***“Epidemiologicy and Infection”***

**Title: *“*Trends of hospitalizations rates in a cohort of HIV-infected persons followed in an Italian hospital from 1998 to 2016**

**Authors** : S. Bellino,

A. Borghetti,

F. Lombardi,

L. Camoni,

A. Ciccullo,

G. Baldin,

S. Belmonti,

D. Moschese,

S. Lamonica,

R. Cauda,

P. Pezzotti,

S. Di Giambenedetto

**Supplementary Table S1**

**Number of total hospitalizations 1998-2016 in each diagnostic category**

| **Diagnostic category** | **Hospitalizations** |
| --- | --- |
| **AIDS-defining diseases** | 1,008 |
| lymphoma non-Hodgkin | 392 |
| Kaposi sarcoma | 207 |
| tuberculosis | 116 |
| mycobacterium avium complex | 97 |
| pneumocystis carinii pneumonia | 59 |
| cryptococcosis | 57 |
| toxoplasmosis of brain | 55 |
| cytomegalovirus disease | 54 |
| progressive multifocal leukoencephalopathy | 37 |
| candidiasis of the esophagus | 35 |
| cervical cancer | 19 |
| encephalopathy HIV-related | 4 |
| other AIDS events | 70 |
| **Non-HIV/AIDS-related infections** | 855 |
| acute and chronic viral hepatitis | 549 |
| non-AIDS parasitosis | 111 |
| sexually transmitted diseases | 73 |
| viral infections | 69 |
| sepsis | 56 |
| bacterial infections | 27 |
| non-AIDS mycosis | 27 |
| viral meningoencephalitis | 9 |
| other unspecified infections | 5 |
| **Non-AIDS-related tumours** | 414 |
| anal cancer | 41 |
| Hodgkin lymphoma | 41 |
| digestive system | 39 |
| malignant secondary tumours | 33 |
| respiratory tract | 30 |
| genitals | 24 |
| melanoma | 16 |
| liver | 15 |
| leukemia | 15 |
| urinary tract | 8 |
| breast | 7 |
| endocrine | 6 |
| other tumours | 31 |
| chemotherapy | 89 |
| benign tumours | 39 |
| **Cardiovascular and other circulatory diseases** | 445 |
| hypertension | 188 |
| atherosclerotic cardiovascular disease | 119 |
| other cardiac pathologies | 88 |
| artery disease | 37 |
| veins, lymph nodes | 33 |
| endocarditis bacterial infections | 32 |
| cardiopulmonary diseases | 23 |
| **Psychiatric disorders** | 371 |
| addiction | 277 |
| depression | 32 |
| psychosis | 31 |
| neurosis and personality disorders | 14 |
| other unspecified disturbances | 33 |
| **Neurological diseases** | 250 |
| non-infectious encephalomyelitis | 86 |
| episodic and paroxysmal disorders | 67 |
| peripheral neuropathy | 36 |
| central nervous system infections | 16 |
| demyelinating diseases | 10 |
| cerebral paralysis | 7 |
| other degenerative pathology | 37 |
| **Respiratory system diseases** | 264 |
| pneumonia | 129 |
| obstructive chronic bronchopneumopathy, asthma, bronchiectasis and chronic obstructive diseases | 79 |
| acute respiratory failure | 44 |
| upper airway diseases | 29 |
| pleural diseases | 10 |
| other lung pathologies | 16 |
| **Kidney and urinary tract diseases** | 198 |
| unspecified pathology | 55 |
| urinary tract infection | 48 |
| genital pathologies | 44 |
| acute or chronic renal failure | 31 |
| urinary tract lithiasis | 24 |
| glomerular disease | 4 |
| **Skin and subcutaneous tissue diseases** | 61 |
| cute infections and soft tissues | 7 |
| other skin disorders | 54 |
| **Other** | 258 |
| ear, nose and throat diseases | 3 |
| haematological | 3 |
| endocrine and metabolic | 3 |
| gastrointestinal | 3 |
| musculoskeletal system and connective tissue | 3 |
| other conditions | 258 |

Reasons for hospitalization were non-mutually exclusive
