# Supplementary material for: Trends of hospitalisations rates in a cohort of HIV-infected persons followed in an Italian hospital from 1998 to 2016
Source: Epidemiol Infect. 2019 Feb 22;147:e89. doi: 10.1017/S0950268819000098 (PMC6518563; doi:10.1017/S0950268819000098)
Supplement: Supplementary file 1 [file S0950268819000098sup.zip › S0950268819000098sup002.doc]

Supplementary Table S2. Incidence rate ratios for hospitalizations, overall and for major diagnostic groups per period

| **Causes of hospitalization** | **IRR (95% CI)** | ***p-value*** | ***Chi-square test*** |
| --- | --- | --- | --- |
| **Total hospitalizations** |  |  |  |
| **All causes** |  |  |  |
| 1998-2000 | 1.00 |  |  |
| 2001-2004 | 0.69 (0.60-0.80) | ***<0.0001*** |  |
| 2005-2008 | 0.45 (0.38-0.52) | ***<0.0001*** |  |
| 2009-2012 | 0.26 (0.22-0.31) | ***<0.0001*** |  |
| 2013-2016 | 0.18 (0.15-0.22) | ***<0.0001*** | ***<0.0001*** |
| **AIDS-defining diseases** |  |  |  |
| 1998-2000 | 1.00 |  |  |
| 2001-2004 | 0.65 (0.50-0.85) | *0.0014* |  |
| 2005-2008 | 0.28 (0.21-0.38) | ***<0.0001*** |  |
| 2009-2012 | 0.10 (0.07-0.15) | ***<0.0001*** |  |
| 2013-2016 | 0.04 (0.03-0.07) | ***<0.0001*** | ***<0.0001*** |
| **Non-HIV/AIDS-related infections** |  |  |  |
| 1998-2000 | 1.00 |  |  |
| 2001-2004 | 1.23 (0.93-1.63) | *0.1423* |  |
| 2005-2008 | 0.70 (0.52-0.94) | ***0.0200*** |  |
| 2009-2012 | 0.45 (0.33-0.62) | ***<0.0001*** |  |
| 2013-2016 | 0.21 (0.15-0.30) | ***<0.0001*** | ***<0.0001*** |
| **Non-AIDS-related tumours** |  |  |  |
| 1998-2000 | 1.00 |  |  |
| 2001-2004 | 0.92 (0.58-1.47) | *0.7402* |  |
| 2005-2008 | 1.15 (0.72-1.84) | *0.5632* |  |
| 2009-2012 | 1.05 (0.64-1.72) | *0.8434* |  |
| 2013-2016 | 0.76 (0.45-1.29) | *0.3116* | *0.2217* |
| **Cardiovascular and other circulatory diseases** |  |  |  |
| 1998-2000 | 1.00 |  |  |
| 2001-2004 | 1.05 (0.64-1.71) | *0.8498* |  |
| 2005-2008 | 2.40 (1.47-3.91) | ***0.0005*** |  |
| 2009-2012 | 1.96 (1.18-3.26) | ***0.0096*** |  |
| 2013-2016 | 1.92 (0.14-3.23) | ***0.0135*** | ***<0.0001*** |
| **Psychological disorders** |  |  |  |
| 1998-2000 | 1.00 |  |  |
| 2001-2004 | 0.89 (0.62-1.26) | *0.5076* |  |
| 2005-2008 | 1.06 (0.70-1.61) | *0.7781* |  |
| 2009-2012 | 0.41 (0.25-0.66) | ***0.0002*** |  |
| 2013-2016 | 0.12 (0.07-0.22) | ***<0.0001*** | ***<0.0001*** |
| **Neurological diseases** |  |  |  |
| 1998-2000 | 1.00 |  |  |
| 2001-2004 | 0.55 (0.35-0.88) | ***0.0127*** |  |
| 2005-2008 | 0.42 (0.26-0.70) | ***0.0007*** |  |
| 2009-2012 | 0.17 (0.09-0.29) | ***<0.0001*** |  |
| 2013-2016 | 0.12 (0.07-0.23) | ***<0.0001*** | ***<0.0001*** |
| **Respiratory tract diseases** |  |  |  |
| 1998-2000 | 1.00 |  |  |
| 2001-2004 | 0.38 (0.22-0.68) | ***0.0010*** |  |
| 2005-2008 | 0.58 (0.33-1.00) | *0.0519* |  |
| 2009-2012 | 0.32 (0.18-0.57) | ***0.0011*** |  |
| 2013-2016 | 0.44 (0.25-0.78) | ***0.0047*** | ***<0.0001*** |
| **Kidney and urinary tract diseases** |  |  |  |
| 1998-2000 | 1.00 |  |  |
| 2001-2004 | 0.52 (0.27-1.03) | *0.0596* |  |
| 2005-2008 | 0.69 (0.36-1.32) | *0.2631* |  |
| 2009-2012 | 1.19 (0.63-2.24) | *0.5830* |  |
| 2013-2016 | 0.60 (0.31-1.20) | *0.1520* | ***0.0006*** |
| **Skin and subcutaneous tissue diseases** |  |  |  |
| 1998-2000 | 1.00 |  |  |
| 2001-2004 | 0.52 (0.21-1.32) | *0.1703* |  |
| 2005-2008 | 0.53 (0.21-1.34) | *0.1814* |  |
| 2009-2012 | 0.31 (0.12-0.83) | ***0.0198*** |  |
| 2013-2016 | 0.25 (0.09-0.68) | ***0.0066*** | *0.0566* |
| **Other** |  |  |  |
| 1998-2000 | 1.00 |  |  |
| 2001-2004 | 0.42 (0.23-0.75) | ***0.0033*** |  |
| 2005-2008 | 0.24 (0.13-0.44) | ***<0.0001*** |  |
| 2009-2012 | 0.17 (0.09-0.31) | ***<0.0001*** |  |
| 2013-2016 | 0.07 (0.04-0.15) | ***<0.0001*** | ***<0.0001*** |

CI, confidence interval; IR, incidence rate, IRR, incidence rate ratio; IRR and p-value were estimated using the random-effects Poisson regression model. Reasons for hospitalization were non-mutually exclusive.

"Other" category includes signs and symptoms classified elsewhere and factors influencing health status; three hospitalizations due to ear-nose-mouth, haematological, endocrine-metabolic, gastrointestinal and musculoskeletal diseases were also included.
