# Supplementary material for: Trends of hospitalisations rates in a cohort of HIV-infected persons followed in an Italian hospital from 1998 to 2016
Source: Epidemiol Infect. 2019 Feb 22;147:e89. doi: 10.1017/S0950268819000098 (PMC6518563; doi:10.1017/S0950268819000098)
Supplement: Supplementary file 1 [file S0950268819000098sup.zip › S0950268819000098sup003.doc]

**Table S3a. Multivariable analysis of factors associated with hospitalization (model with ART effect, without CD4 and Viral load effects**)

|  | **All Hospitalizations** | |
| --- | --- | --- |
| **Factors** | **RR (95% CI)** | ***p-value*** |
| **Class of Age (years)** |  |  |
| <35 | 1.00 | *(ref)* |
| 35-44 | 1.11 (0.96-1.29) | *0.1750* |
| 45-54 | 1.13 (0.93-1.38) | *0.2103* |
| ≥55 | 2.16 (1.65-2.83) | ***<0.0001*** |
| **Gender** |  |  |
| Male | 1.00 | *(ref)* |
| Female | 1.43 (1.11-1.83) | ***0.0055*** |
| **HIV exposure** |  |  |
| Heterosexual contact | 1.00 | *(ref)* |
| Homosexual contact | 1.02 (0.78-1.33) | *0.8627* |
| Injection drug use | 1.32 (0.98-1.78) | *0.0693* |
| Not reported | 3.23 (2.15-4.84) | ***<0.0001*** |
| **Years since first positive HIV test** |  |  |
| 0-4 | 1.00 | *(ref)* |
| 5-10 | 1.13 (1.01-1.27) | ***0.0307*** |
| >10 | 1.68 (1.41-1.99) | ***<0.0001*** |
| **Hepatitis C virus coinfection** | 1.23 (1.03-1.47) | ***0.0237*** |
| **CD4 nadir (cells/µl)** |  |  |
| <200 | 1.00 | *(ref)* |
| 200-349 | 0.43 (0.36-0.52) | ***<0.0001*** |
| ≥350 | 0.24 (0.19-0.29) | ***<0.0001*** |
| **Antiretroviral therapy** |  |  |
| Naive | 1.00 | *(ref)* |
| On ART | 0.47 (0.42-0.54) | ***<0.0001*** |
| **Period** | 1.00 | *(ref)* |
| 1998-2000 | 0.47 (0.42-0.54) | ***<0.0001*** |
| 2001-2004 | 0.65 (0.56-0.76) | ***<0.0001*** |
| 2005-2008 | 0.38 (0.32-0.45) | ***<0.0001*** |
| 2009-2012 | 0.20 (0.17-0.25) | ***<0.0001*** |
| 2013-2016 | 0.13 (0.10-0.16) | ***<0.0001*** |

**Table S3b. Multivariable analysis of factors associated with hospitalization (model with CD4 and viral load effects, without ART effect**)

|  | **All Hospitalizations** | |
| --- | --- | --- |
| **Factors** | **RR (95% CI)** | ***p-value*** |
| **Class of Age (years)** |  |  |
| <35 | 1.00 | *(ref)* |
| 35-44 | 1.10 (0.95-1.27) | *0.2183* |
| 45-54 | 1.20 (0.99-1.46) | *0.0590* |
| ≥55 | 2.36 (1.83-3.07) | ***<0.0001*** |
| **Gender** |  |  |
| Male | 1.00 | *(ref)* |
| Female | 1.46 (1.16-1.84) | ***0.0015*** |
| **HIV exposure** |  |  |
| Heterosexual contact | 1.00 | *(ref)* |
| Homosexual contact | 1.03 (0.80-1.32) | *0.8341* |
| Injection drug use | 1.29 (0.97-1.70) | *0.0764* |
| Not reported | 2.69 (1.83-3.93) | ***<0.0001*** |
| **Years since first positive HIV test** |  |  |
| 0-4 | 1.00 | *(ref)* |
| 5-10 | 1.22 (1.09-1.38) | ***0.0008*** |
| >10 | 1.69 (1.42-2.00) | ***<0.0001*** |
| **Hepatitis C virus coinfection** | 1.27 (1.07-1.52) | ***0.0076*** |
| **CD4 nadir (cells/µl)** |  |  |
| <200 | 1.00 | *(ref)* |
| 200-349 | 0.86 (0.71-1.04) | ***0.1218*** |
| ≥350 | 0.63 (0.50-0.79) | ***0.0001*** |
| **CD4 (cells/µl), HIV RNA (copies/ml), Period** |  |  |
| **CD4 <200, HIV RNA >50, 1998-2000** | 1.00 | *(ref)* |
| CD4 <200, VL >50, 2001-2004 | 1.28 (0.99-1.65) | *0.0564* |
| CD4 <200, VL >50, 2005-2008 | 0.93 (0.69-1.25) | *0.6286* |
| CD4 <200, VL >50, 2009-2012 | 0.56 (0.39-0.81) | ***0.0019*** |
| CD4 <200, VL >50, 2013-2016 | 0.47 (0.30-0.74) | ***0.0012*** |
| **CD4 200-349, HIV RNA >50, 1998-2000** | 1.00 | *(ref)* |
| CD4 200-349, VL >50, 2001-2004 | 0.56 (0.39-0.80) | ***0.0018*** |
| CD4 200-349, VL >50, 2005-2008 | 0.53 (0.36-0.77) | ***0.0010*** |
| CD4 200-349, VL >50, 2009-2012 | 0.24 (0.16-0.38) | ***<0.0001*** |
| CD4 200-349, VL >50, 2013-2016 | 0.14 (0.08-0.26) | ***<0.0001*** |
| **CD4 350-499, HIV RNA >50, 1998-2000** | 1.00 | *(ref)* |
| CD4 350-499, VL >50, 2001-2004 | 1.09 (0.68-1.73) | *0.7262* |
| CD4 350-499, VL >50, 2005-2008 | 0.91 (0.56-1.47) | *0.7000* |
| CD4 350-499, VL >50, 2009-2012 | 0.40 (0.23-0.71) | ***0.0015*** |
| CD4 350-499, VL >50, 2013-2016 | 0.25 (0.13-0.49) | ***<0.0001*** |
| **CD4 ≥500, HIV RNA >50, 1998-2000** | 1.00 | *(ref)* |
| CD4 ≥500, VL >50, 2001-2004 | 0.55 (0.36-0.84) | ***0.0056*** |
| CD4 ≥500, VL >50, 2005-2008 | 0.27 (0.17-0.43) | ***<0.0001*** |
| CD4 ≥500, VL >50, 2009-2012 | 0.23 (0.14-0.37) | ***<0.0001*** |
| CD4 ≥500, VL >50, 2013-2016 | 0.09 (0.05-0.16) | ***<0.0001*** |
| **CD4 <200, HIV RNA ≤50, 1998-2000** | 1.00 | *(ref)* |
| CD4 <200, VL ≤50, 2001-2004 | 0.61 (0.41-0.93) | ***0.0200*** |
| CD4 <200, VL ≤50, 2005-2008 | 0.29 (0.18-0.45) | ***<0.0001*** |
| CD4 <200, VL ≤50, 2009-2012 | 0.19 (0.11-0.31) | ***<0.0001*** |
| CD4 <200, VL ≤50, 2013-2016 | 0.29 (0.17-0.49) | ***<0.0001*** |
| **CD4 200-349, HIV RNA ≤50, 1998-2000** | 1.00 | *(ref)* |
| CD4 200-349, VL ≤50, 2001-2004 | 0.29 (0.18-0.48) | ***<0.0001*** |
| CD4 200-349, VL ≤50, 2005-2008 | 0.17 (0.11-0.28) | ***<0.0001*** |
| CD4 200-349, VL ≤50, 2009-2012 | 0.16 (0.10-0.26) | ***<0.0001*** |
| CD4 200-349, VL ≤50, 2013-2016 | 0.08 (0.05-0.13) | ***<0.0001*** |
| **CD4 350-499, HIV RNA ≤50, 1998-2000** | 1.00 | *(ref)* |
| CD4 350-499, VL ≤50, 2001-2004 | 0.82 (0.36-1.87) | *0.6409* |
| CD4 350-499, VL ≤50, 2005-2008 | 0.66 (0.30-1.47) | *0.3147* |
| CD4 350-499, VL ≤50, 2009-2012 | 0.56 (0.26-1.24) | *0.1549* |
| CD4 350-499, VL ≤50, 2013-2016 | 0.39 (0.17-0.87) | ***0.0208*** |
| **CD4 ≥500, HIV RNA ≤50, 1998-2000** | 1.00 | *(ref)* |
| CD4 ≥500, VL ≤50, 2001-2004 | 0.29 (0.15-0.56) | ***0.0002*** |
| CD4 ≥500, VL ≤50, 2005-2008 | 0.23 (0.12-0.42) | ***<0.0001*** |
| CD4 ≥500, VL ≤50, 2009-2012 | 0.14 (0.07-0.25) | ***<0.0001*** |
| CD4 ≥500, VL ≤50, 2013-2016 | 0.10 (0.05-0.19) | ***<0.0001*** |
